# Eight‐year outcomes of aortic valve replacement with the Carpentier‐Edwards PERIMOUNT Magna Ease valve

**DOI:** 10.1111/jocs.17140

**Published:** 2022-11-15

**Authors:** Steven Tsui, Michael Rosenbloom, James Abel, Jeffrey Swanson, Axel Haverich, Joseph Zacharias, Gilbert Schorlemmer, Gideon Cohen, Michael Moulton, Rüdiger Lange

**Affiliations:** ^1^ Department of Cardiothoracic Surgery and Transplantation Royal Papworth Hospital Cambridge UK; ^2^ Division of Cardiothoracic Surgery Cooper University Hospital Camden New Jersey USA; ^3^ Division of Cardiac and Thoracic Surgery, St Paul's Hospital University of British Columbia Vancouver British Columbia Canada; ^4^ Providence Heart Valve Clinic Providence St Vincent's Hospital Portland Oregon USA; ^5^ Department of Cardiothoracic, Transplantation and Vascular Surgery Medizinische Hochschule Hannover Hannover Germany; ^6^ Department of Cardiothoracic Surgery Blackpool Victoria Hospital Blackpool UK; ^7^ Department of Cardiac, Vascular and Thoracic Surgery St Mark's Hospital Salt Lake Utah USA; ^8^ Department of Surgery, Division of Cardiac Surgery Sunnybrook Health Sciences Center North York Ontario Canada; ^9^ Division of Cardiothoracic Surgery University of Nebraska Medical Center Omaha Nebraska USA; ^10^ Department of Cardiovascular Surgery German Heart Center Munich Munich Bavaria Germany

**Keywords:** aortic stenosis, aortic valve replacement, bioprosthesis, bioprosthetic valve

## Abstract

**Introduction:**

The Carpentier‐Edwards PERIMOUNT Magna Ease valve is a third‐generation bioprosthesis for aortic valve replacement (AVR). This is a postapproval study reporting on its 8‐year outcomes.

**Methods:**

Adults undergoing AVR with the Magna Ease valve between October 2007 and December 2012 were enrolled for this prospective, nonrandomized, single‐arm, and multicenter study. Assessments occurred preoperatively, at hospital discharge, 6 months, 1 year, and annually thereafter for up to 8 years. Outcomes included safety endpoints, hemodynamic performance, and New York Heart Association (NYHA) functional class.

**Results:**

Of the 258 study patients, 67.5% were in NYHA Class I or II, and 32.5% were in NYHA Class III or IV at baseline. Concomitant procedures were performed in 44.2%. Total follow‐up was 1597.6 patient‐years, and median follow‐up was 7 years (interquartile range: 5.5–8.0 years). Eight years following AVR, the functional class remained improved from baseline with 93.9% in NYHA Class I/II and 6.1% in NYHA Class III; 38 deaths had occurred, 8 of which were valve related; freedom from all‐cause mortality was 80.7% (95% confidence intervals: 74.9, 86.4); freedom from valve‐related mortality was 95.8% (92.8, 98.8); freedom from reintervention, explant, major bleeding events, and structural valve deterioration was 89.8% (85.1, 94.6), 94.8% (91.7, 97.9), 85.1% (80.0, 90.1), and 90.1% (84.7, 95.4), respectively; effective orifice area was 1.5 ± 0.5 cm^2^, the mean gradient was 14.8 ± 8.3 mmHg, and 88.6% of patients had no or trivial aortic regurgitation.

**Conclusions:**

This study demonstrated satisfactory safety and sustained hemodynamic and functional improvements at 8 years following AVR with the Magna Ease valve.

**Abbreviations**
AVR: aortic valve replacement
CEC: Clinical Events Committee
CI: confidence interval
NSVD: nonstructural valve dysfunction
NYHA: New York Heart Association
SVD: structural valve deterioration

## 1 INTRODUCTION

Stenosis and regurgitation are common conditions affecting the aortic valve.^1^ When severe and symptomatic, aortic valve replacement (AVR) is the guideline‐recommended treatment.^2^, ^3^ Over the last 2 decades, the proportion of AVR undertaken with bioprostheses has increased, substituting for mechanical valve prostheses.^4^

The effectiveness and durability of the Carpentier‐Edwards PERIMOUNT valve (Edwards Lifesciences), a stented bovine pericardial bioprosthesis, have been well described.^5^, ^6^, ^7^, ^8^ The Carpentier‐Edwards PERIMOUNT Magna Ease aortic valve (model 3300TFX; Edwards Lifesciences) is an evolution of the original PERIMOUNT valve. The reduced profile was designed to facilitate supra‐annular placement. It also incorporates a scalloped sewing ring to improve conformity with the native aortic leaflet attachment line. The Magna Ease valve was approved by the United States (US) Food and Drug Administration (FDA) in 2009. In a single‐center study, it demonstrated excellent midterm survival and good hemodynamics, but additional durability data are required.^9^ This study was conducted to satisfy conditional FDA approval, evaluating the 8‐year safety and effectiveness of the Magna Ease valve in patients undergoing AVR with or without concomitant procedures.

## 2 METHODS

### 2.1 Study design

This study was a prospective, nonrandomized, single‐arm, postapproval, multicenter, 8‐year study of the Magna Ease valve (ClinicalTrials.gov NCT01171625). The FDA required data from at least 101 patients over 8 years. The study protocol projected that at least 225 patients would need to be enrolled at the beginning to achieve the required number at an 8‐year follow‐up and the study was terminated when this number was reached. The study protocol complied with ISO 14155:2011; European Medical Device Directive 2007/47/EC; and MedDev 2.12‐1, 2.7.4, and 2.12.2. The ICH E6 GCP Good Clinical Practices was also used for guidance.

### 2.2 Study cohort

**Figure 1:**
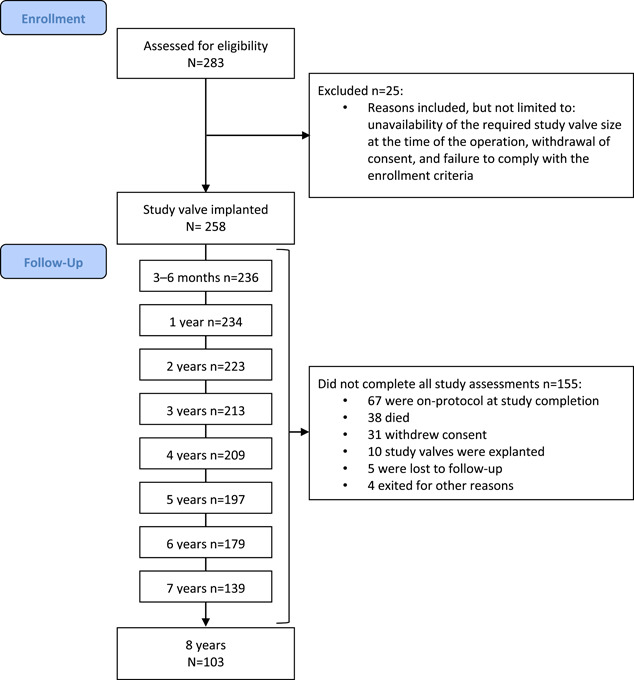
Consolidated standards of reporting trials flow diagram

Patients were enrolled between October 2007 and December 2012 at 14 investigational sites in Europe, Canada, and the US (Figure 1). Patients undergoing surgical replacement of their native or prosthetic aortic valve at participating centers were invited to participate. The inclusion criteria for this study were: requirement for a replacement aortic valve, as indicated in the preoperative evaluation; average or better operative risk; geographically stable and agreeable to attend follow‐up assessments at the hospital of surgical services for at least 8 years; 18 years or older; signed and dated the subject informed consent form before surgery. The study's exclusion criteria were: any known noncardiac life‐threatening disease, which will limit the patient's life expectancy below 1 year; active endocarditis within the last 3 months; abnormal calcium metabolism (e.g., chronic renal failure, hyperparathyroidism); aneurismal aortic degenerative condition (e.g., cystic medial necrosis, Marfan's syndrome); pregnant or lactating; intravenous drug abuse; current prison inmate; current participant in a study of an investigational drug or device; requirement for replacement of a native or prosthetic mitral, tricuspid, or pulmonic valve; requirement for the repair of the mitral or tricuspid valve with the use of an annuloplasty device; previous enrolment in the study; prior mitral, tricuspid, or pulmonic valve surgery, which included implantation of a bioprosthetic valve, mechanical valve, or annuloplasty ring that will remain in situ. The choice of surgical technique was left to surgeon's discretion.

### 2.3 Follow‐up and endpoints

After implantation, patients were followed up at hospital discharge, 6 months, 1 year, and annually thereafter for up to 8 years. This report includes data through to September 14, 2018, when the minimum requirement of 8‐year follow‐up for 101 patients had been achieved.

Safety endpoints included death, valve‐related death, thromboembolism, hemorrhage, paravalvular leak, prosthetic valve endocarditis, valve thrombosis, hemolysis, structural valve deterioration (SVD), nonstructural valve dysfunction (NSVD), reintervention, and valve explant. All safety endpoints and serious adverse events were defined as per Akins et al.^10^ and adjudicated by an independent Clinical Events Committee (CEC).

Effectiveness endpoints included the proportion of patients in New York Heart Association (NYHA) Functional Class I or II at 8 years, and hemodynamic performance as assessed by echocardiographic parameters: effective orifice area, mean gradient, and aortic regurgitation (combined paravalvular and central leak) severity. Hemodynamic data were collected and scored by the individual sites. Collection of these data in Years 3 and 7 was not mandated and therefore not reported.

### 2.4 Data management and statistical analyses

The investigational sites were responsible for the accurate collection and recording of the clinical data. Edwards Lifesciences, the study sponsor, monitored and aggregated the clinical data, then analyzed them per the study protocol and statistical analysis plan. Summary statistics include absolute and proportional data for categorical variables and mean ± standard deviation for continuous variables. Early safety events were defined as those occurring within 30 days of the index procedure and were reported as the number of patients with an event divided by the number of implanted patients. Late events represented those occurring beyond 30 days postoperatively and through 8 years (postoperative days 2922). Actuarial Kaplan–Meier analyses were undertaken on each of the safety endpoints and reported with 95% confidence intervals (CIs). SAS version 9.4 was used for all statistical analyses.

## 3 RESULTS

### 3.1 Baseline patient characteristics

A total of 283 patients across 14 investigational sites consented to participate. Of these, 258 patients met the eligibility criteria and received the Magna Ease valve, the outcomes of whom are reported here. Table 1 summarizes patient baseline characteristics. The average age was 68.5 ± 8.8 years, and 167 (64.7%) were male. Aortic stenosis was the commonest indication for AVR (70.9%). Procedural data are summarized in Table 2. Most patients underwent isolated AVR (58.1%), while 41.9% underwent the concomitant procedure(s). Implanted valve sizes were: 19 mm 4.3%, 21 mm 15.9%, 23 mm 38.4%, 25 mm 28.3%, 27 mm 8.9%, and 29 mm 4.3%.

**Table 1:** Baseline characteristics

Characteristic	*n*	% (*n/N*)
Sex		
Female	91	35.3
Male	167	64.7
Age, years ± SD (range)	68.5 ± 8.8 (36.1–86.4)	–
Body mass index, kg/m^2^ ± SD (range)	28.9 ± 5.6 (18.1–50.8)	–
NYHA classification		
I	40	15.9
II	130	51.6
III	76	30.2
IV	6	2.4
Comorbidities		
Hypertension	168	65.1
Hyperlipidemia/Hypercholesterolemia	163	63.2
Coronary artery disease	106	41.1
Diabetes	56	21.7
Pulmonary disease	29	11.2
Smoking (current)	16	6.2
TIA/CVA	15	5.8
Liver disease	7	2.7
Endocarditis	5	1.9
Renal failure	5	1.9
Prior cardiovascular interventions		
Aortic valve replacement	2	0.8
CABG	2	0.8
Pacemaker implant	1	0.4
Aortic valve disease etiology^a^		
Calcified	177	68.6
Degenerative	81	31.4
Congenital	52	20.2
Rheumatic	14	5.4
Remote endocarditis	9	3.5
Other^b^	8	3.1

Abbreviations: CABG, coronary artery bypass graft; CVA, cerebrovascular accident; NYHA, New York Heart Association; SD, standard deviation; TIA, transient ischemic attack.

^a^ Patients may present with more than one type of disease etiology.

^b^ Includes perforation of the white coronary cusp of the aortic valve and stenosis/regurgitation.

**Table 2:** Procedural data

Concomitant procedures^a^	*n*	% (*n/N*)
None	150	58.1
CABG	71	27.5
Myectomy	32	12.4
Ascending aortic aneurysm repair	15	5.8
Occlusion of the left atrial appendage	4	1.6
Ablation	3	1.2
Other^b^	8	3.1

Abbreviation: CABG, coronary artery bypass graft.

^a^ More than one type of concomitant procedure per patient is possible.

^b^ Other procedures include aortic root enlargement, aortoplasty, aortic root replacement, left ventricle tumor excision, and maze procedure.

### 3.2 Patient follow‐up

Overall, 258 patients underwent a total follow‐up of 1597.6 patient‐years: median 7.0 years (5.5–8.0 years). At study closure, 103 patients (39.9%) had completed the 8‐year follow‐up and a further 67 patients (26.0%) were on protocol with the study valve in place.

### 3.3 Survival

**Figure 2:**
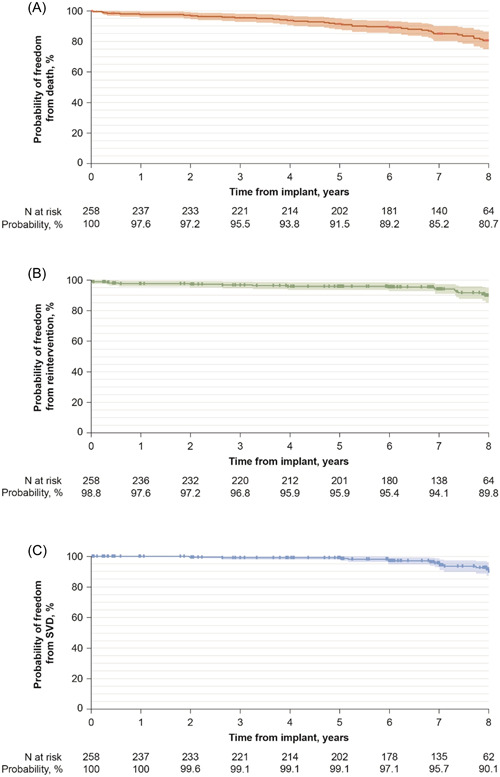
Kaplan–Meier survival curves (solid lines) and 95% confidence intervals (shaded bands) for freedom from all‐cause mortality (A), reintervention (B), and SVD (C). SVD, structural valve deterioration.

One death (0.4%) occurred within the early postoperative period, and it was not valve related. Thirty‐seven deaths occurred late, eight of which were adjudicated to be valve related: two endocarditis, one thromboembolism/stroke, and five other unknown causes. Figure 2 and Table 3 display the freedom from all‐cause mortality.

**Table 3:** Actuarial freedom from safety events for all patients based on Kaplan–Meier analyses

		Freedom from event					
		1 year		5 years		8 years	
Adverse event or outcome	Early *n*, *m*, (% [*n/N*])	#At risk #Events	Event free, % (95% CI)	#At risk #Events	Event free, % (95% CI)	#At risk #Events	Event free, % (95% CI)
Death	1, 1 (0.4)	237	97.6	202	91.5	64	80.7
		6	(95.7, 99.5)	20	(88.0, 95.1)	38	(74.9, 86.4)
Device‐related death	0, 0 (0.0)	237	99.6	202	97.8	64	95.8
		1	(98.8, 100.0)	5	(95.9, 99.7)	8	(92.8, 98.8)
Reintervention	3,3 (1.2)	236	97.6	201	95.9	64	89.8
		6	(95.8, 99.5)	10	(93.4, 98.4)	18	(85.1, 94.6)
Explant	3, 3 (1.2)	237	98.0	202	96.3	64	94.8
		5	(96.3, 99.7)	9	(93.9, 98.7)	11	(91.7, 97.9)
Bleeding event	15, 16 (6.2)	212	88.4	168	81.8	48	78.0
		29	(84.5, 92.4)	44	(76.9, 86.7)	49	(72.2, 83.7)
Major bleeding event	12, 13 (5.0)	221	92.5	181	88.9	53	85.1
		19	(89.3, 95.8)	27	(84.9, 92.9)	32	(80.0, 90.1)
Thromboembolism	5, 5 (1.9)	227	96.0	186	90.7	58	86.7
		10	(93.6, 98.4)	22	(87.0, 94.4)	28	(81.9, 91.5)
Endocarditis	0, 0 (0.0)	237	98.8	202	97.9	64	97.3
		3	(97.5, 100.0)	5	(96.1, 99.7)	6	(95.1, 99.5)
SVD	0, 0 (0.0)	237	100.0	202	99.1	62	90.1
		0	(100.0, 100.0)	2	(97.9, 100.0)	14	(84.7, 95.4)
Reintervention due to SVD	0, 0 (0.0)	237	100.0	202	99.1	64	93.6
		0	(100.0, 100.0)	2	(97.9, 100.0)	9	(89.3, 97.8)
Hemolysis	0, 0 (0.0)	235	99.2	200	99.2	64	99.2
		2	(98.0, 100.0)	2	(98.0, 100.0)	2	(98.0, 100.0)
Nonstructural valve dysfunction	1, 1 (0.4)	235	98.8	199	97.9	63	97.9
		3	(97.4, 100.0)	5	(96.1, 99.7)	5	(96.1, 99.7)
Paravalvular leak	1, 1 (0.4)	235	98.8	199	98.4	63	98.4
		3	(97.4, 100.0)	4	(96.8, 100.0)	4	(96.8, 100.0)
Major paravalvular leak	1, 1 (0.4)	236	99.2	201	99.2	64	99.2
		2	(98.1, 100.0)	2	(98.1, 100.0)	2	(98.1, 100.0)
Valve thrombosis	0, 0 (0.0)	237	100.0	202	100.0	64	100.0
		0	(100.0, 100.0)	0	(100.0, 100.0)	0	(100.0, 100.0)

*Note*: “*m*” is the number of events, “*n*” is the number of patients with an event. Survival estimates and 95% CIs based on Kaplan–Meier analysis of time to the first occurrence (early or late).

Abbreviations: CI, confidence interval; SVD, structural valve deterioration.

### 3.4 Safety

Safety endpoint events were experienced by 109 (42.2%) patients and are summarized in Table 3 as early event rates and actuarial freedom from safety endpoints based on Kaplan–Meier analyses.

Study valve reinterventions were carried out in 18 patients, including 11 explants, 6 valve‐in‐valve insertions, and 1 repair procedure without explant. There were three early reinterventions, all explants that occurred during the index procedure due to complications during surgery not related to the study valve. The reasons for the 15 late reinterventions were endocarditis (*n* = 4), SVD (*n* = 9), NSVD (*n* = 1), and major paravalvular leak (*n* = 1). Freedom from study valve reintervention was 89.8% (95% CI: 85.1, 94.6) at 8 years. Freedom from explant was 94.8% (95% CI: 91.7, 97.9) at 8 years.

In the early period, 13 major bleeding events were reported in 12 patients and 3 minor bleeding events were reported in 3 patients. A further 19 major bleeding events and 14 minor bleeding events were reported in the late period through to 8 years. No bleeding events were adjudicated as study valve related. Six cases of prosthetic valve endocarditis were reported in six patients. Four of these resulted in study valve explant followed by one death, and one further case resulted in death, all of which were adjudicated to be study valve related. The remaining case had high‐grade lactobacillus bacteremia and was categorized as valve related. This patient proceeded to develop SVD and the study valve was explanted. Freedom from bleeding events was 78.0% (95% CI: 72.2, 83.7) at 8 years; freedom from major bleeding events was 85.1% (95% CI: 80.0, 90.1) at 8 years.

### 3.5 Hemodynamics

**Figure 3:**
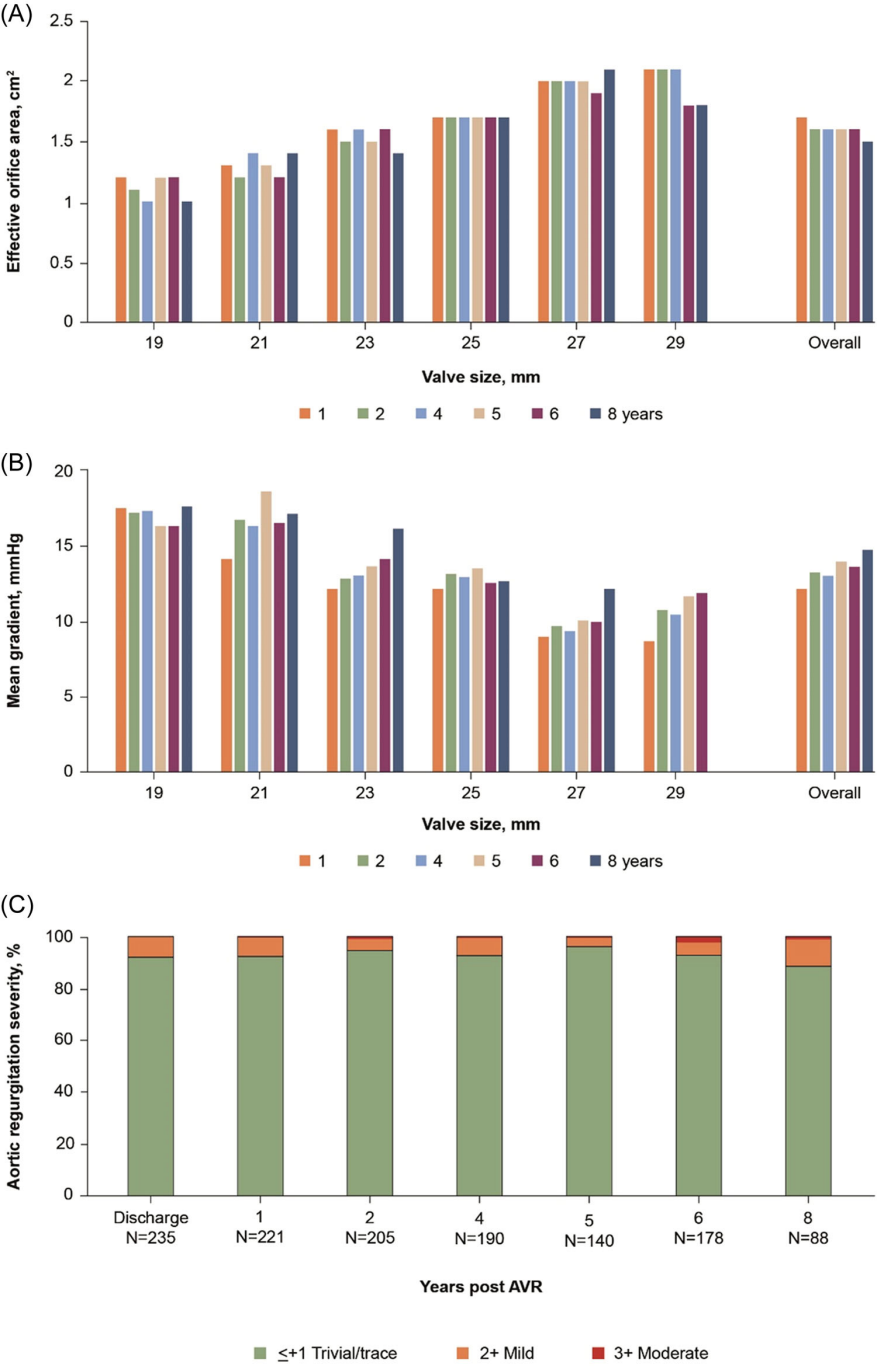
Hemodynamic performance of the Magna Ease valve measured by EOA (A) mean gradient (B) and severity of aortic regurgitation (C). AVR, aortic valve replacement; EOA, effective orifice area.

Figure 3 and Table 4 show the hemodynamic performance for each study valve size; the performance stayed within expected levels over the observational period. Data on aortic regurgitation are shown in Table 5.

**Table 4:** Hemodynamic performance by valve size

Visit	19 mm	21 mm	23 mm	25 mm	27 mm		29 mm		Overall	
	*n*	*n*	*n*	*n*	*n*		*n*		*n*	
	Mean ± SD	Mean ± SD	Mean ± SD	Mean ± SD	Mean ± SD		Mean ± SD		Mean ± SD	
	Median, Q1, Q3	Median, Q1, Q3	Median, Q1, Q3	Median, Q1, Q3	Median, Q1, Q3		Median, Q1, Q3		Median, Q1, Q3	
*Aortic EOA*, cm^2^										
Discharge	9	34	87	66	19		10		225	
	1.3 ± 0.37	1.5 ± 0.42	1.7 ± 0.36	1.9 ± 0.59	2.3 ± 0.67		2.5 ± 0.61		1.8 ± 0.55	
	1.2, 1.1, 1.3	1.4, 1.1, 1.7	1.6, 1.4, 1.9	1.8, 1.5, 2.1	2.1, 1.9, 2.6		2.4, 2.2, 2.8		1.7, 1.4, 2.0	
1 year	7	28	82	59	19		10		205	
	1.2 ± 0.41	1.3 ± 0.29	1.6 ± 0.37	1.7 ± 0.36	2.0 ± 0.31		2.1 ± 0.28		1.7 ± 0.41	
	1.1, 0.9, 1.2	1.3, 1.1, 1.5	1.6, 1.4, 1.8	1.7, 1.5, 2.0	1.9, 1.8, 2.2		2.2, 1.9, 2.4		1.6, 1.4, 1.9	
2 years	6	27	77	58	19		10		197	
	1.1 ± 0.40	1.2 ± 0.30	1.5 ± 0.41	1.7 ± 0.33	2.0 ± 0.31		2.1 ± 0.42		1.6 ± 0.44	
	1.0, 0.8, 1.3	1.3, 1.0, 1.4	1.5, 1.3, 1.7	1.7, 1.5, 1.9	2.1, 1.8, 2.3		2.1, 1.8, 2.3		1.6, 1.3, 1.9	
4 years	5	23	74	56	18		9		185	
	1.0 ± 0.22	1.4 ± 0.31	1.6 ± 0.35	1.7 ± 0.46	2.0 ± 0.52		2.1 ± 0.49		1.6 ± 0.46	
	1.0, 0.9, 1.1	1.3, 1.1, 1.6	1.6, 1.3, 1.8	1.6, 1.4, 1.9	1.9, 1.7, 2.2		1.8, 1.7, 2.5		1.6, 1.3, 1.9	
5 years	3	20	50	43	15		7		138	
	1.2 ± 0.70	1.3 ± 0.32	1.5 ± 0.34	1.7 ± 0.29	2.0 ± 0.38		1.8 ± 0.28		1.6 ± 0.39	
	0.8, 0.8, 2.0	1.2, 1.1, 1.5	1.5, 1.3, 1.7	1.7, 1.5, 1.8	2.1, 1.7, 2.3		1.8, 1.6, 1.9		1.6, 1.3, 1.8	
6 years	8	26	70	47	17		7		175	
	1.2 ± 0.38	1.2 ± 0.39	1.6 ± 0.42	1.7 ± 0.34	1.9 ± 0.44		1.8 ± 0.15		1.6 ± 0.44	
	1.2, 0.8, 1.4	1.2, 0.9, 1.5	1.5, 1.3, 1.8	1.7, 1.4, 1.8	1.9, 1.6, 2.2		1.9, 1.7, 2.0		1.6, 1.3, 1.8	
8 years	3	11	33	34	3		2^a^		86	
	1.0 ± 0.15	1.4 ± 0.40	1.4 ± 0.53	1.7 ± 0.47	2.1 ± 0.32		[1.6, 2.2]		1.5 ± 0.51	
	1.1, 0.8, 1.1	1.2, 1.0, 1.7	1.4, 1.3, 1.6	1.7, 1.3, 1.8	2.0, 1.9, 2.5				1.5, 1.2, 1.7	
*Mean gradient*, mmHg										
Discharge	9	36	91	66	19		11		232	
	19.2 ± 4.72	16.7 ± 6.21	13.8 ± 5.00	13.5 ± 5.42	9.5 ± 3.79		9.4 ± 2.43		13.8 ± 5.56	
	17.9, 16.2, 23.0	15.0, 13.0, 19.5	14.0, 10.0, 17.0	13.0, 10.0, 16.6	9.7, 7.0, 13.0		9.0, 7.0, 12.0		13.0, 10.0, 17.0	
1 year	8	30	90	62	21		10		221	
	17.6 ± 6.98	14.2 ± 5.83	12.2 ± 5.00	12.2 ± 3.69	9.0 ± 3.74		8.7 ± 3.19		12.2 ± 4.97	
	16.1, 13.0, 19.2	13.8, 10.0, 18.7	11.5, 8.2, 15.0	11.8, 9.8, 15.0	8.0, 6.0, 11.0		8.0, 7.0, 10.0		11.1, 9.0, 15.0	
2 years	6	28	79	59	19		10		201	
	17.3 ± 3.59	16.8 ± 6.88	12.9 ± 5.52	13.2 ± 4.13	9.7 ± 3.26		10.8 ± 6.47		13.3 ± 5.50	
	19.0, 14.0, 20.0	16.3, 12.0, 20.5	12.0, 9.0, 15.0	13.0, 10.0, 16.0	9.0, 8.0, 10.0		9.0, 8.0, 10.0		12.0, 9.0, 16.0	
4 years	5	25	75	55	18		9		187	
	17.4 ± 4.75	16.4 ± 6.47	13.1 ± 5.41	13.0 ± 4.40	9.4 ± 3.78		10.5 ± 4.70		13.1 ± 5.38	
	18.0, 17.0, 19.0	16.0, 13.0, 19.4	13.0, 9.0, 16.0	13.0, 9.4, 15.0	9.3, 6.0, 11.0		10.0, 7.0, 11.0		13.0, 9.0, 16.0	
5 years	3	20	51	44	15		7		140	
	16.4 ± 5.34	18.7 ± 6.27	13.7 ± 5.80	13.6 ± 4.68	10.1 ± 4.10		11.7 ± 5.28		14.0 ± 5.73	
	19.0, 10.3, 20.0	17.5, 14.5, 22.5	14.0, 10.0, 16.5	13.0, 10.0, 17.0	9.0, 8.0, 14.0		10.0, 9.0, 15.0		13.8, 10.0, 17.0	
6 years	8	26	70	48	17		7		176	
	16.4 ± 3.63	16.6 ± 7.40	14.2 ± 8.75	12.6 ± 3.48	10.0 ± 4.25		11.9 ± 5.40		13.7 ± 6.92	
	18.0, 13.5, 18.5	15.5, 13.0, 19.0	12.0, 9.0, 17.0	13.0, 10.0, 15.0	10.0, 7.0, 14.0		10.0, 7.0, 17.0		13.0, 9.0, 16.6	
8 years	4	11	33	35	4		2^a^		89	
	17.7 ± 6.38	17.2 ± 10.82	16.2 ± 10.46	12.7 ± 4.56	12.2 ± 4.51		[6.6, 20.0]		14.8 ± 8.26	
	16.9, 13.0, 22.4	16.0, 8.0, 27.0	13.0, 12.0, 17.0	13.0, 9.0, 15.0	13.0, 9.0, 15.3				13.0, 10.0, 17.0	

Abbreviations: EOA, effective orifice area; SD, standard deviation; Q, quartile.

^a^ Where measurements for fewer than three patients were reported, individual values are shown in square parentheses.

**Table 5:** Aortic regurgitation location

	Discharge % (*n/N*) *N* = 235	1 year % (*n/N*) *N* = 221	2 years % (*n/N*) *N* = 205	4 years % (*n/N*) *N* = 190	5 years % (*n/N*) *N* = 140	6 years % (*n/N*) *N* = 178	8 years % (*n/N*) *N* = 88
Paravalvular leak	3.8	2.3	1.0	2.1	2.9	2.8	4.5
	(9/235)	(5/221)	(2/205)	(4/190)	(4/140)	(5/178)	(4/88)
Central leak	10.6	19.5	16.6	23.7	21.4	24.7	21.6
	(25/235)	(43/221)	(34/205)	(45/190)	(30/140)	(44/178)	(19/88)
Indeterminate	6.8	4.5	4.9	3.2	1.4	3.9	6.8
	(16/235)	(10/221)	(10/205)	(6/190)	(2/140)	(7/178)	(6/88)
Not available	–	0.5	–	–	0.7	–	–
		(1/221)			(1/140)		
Paravalvular and central leak	0.4	–	–	–	–	–	–
	(1/235)						

*Note*: *N* is the number of subjects with a valid regurgitation assessment for the specified visit.

### 3.6 Structural valve deterioration

Of the 14 patients with SVD, 3 underwent explant, 7 had valve‐in‐valve procedures, and 4 cases were being monitored at study closure. Freedom from reintervention due to SVD was 93.6% (95% CI: 89.3, 97.8) at 8 years.

### 3.7 Functional outcomes

**Figure 4:**
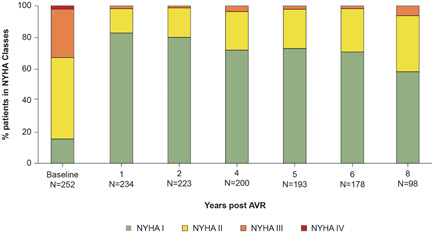
NYHA functional classification assessment of patients following surgical AVR with the Magna Ease valve. AVR, aortic valve replacement; NYHA, New York Heart Association.

Figure 4 shows the proportion of patients in each NYHA Functional Class throughout follow‐up. At baseline, 67.5% of patients were in NYHA Class I/II, and 32.5% of patients were in NYHA Class III/IV. By the 1‐year follow‐up, 98.3% were in Class I/II, with 74.8% of patients reporting an improvement in functional class. This improvement persisted for up to 8 years for 61.9% of patients, with 93.9% of patients in NYHA Class I/II and 6.1% in NYHA Class III.

## 4 DISCUSSION

This is the first prospective evaluation of the Magna Ease bioprosthesis for surgical AVR. These results demonstrate good safety and effectiveness, with functional and hemodynamic outcomes that remain consistent over the 8‐year follow‐up.

### 4.1 Survival

Kaplan–Meier analyses showed freedom from all‐cause and valve‐related mortality at 8 years were 80.7% (95% CI: 74.9, 86.4) and 95.8% (95% CI: 92.8, 98.8), respectively. A recent retrospective study of AVR with the Magna Ease valve, with a median follow‐up of 4.5 years, revealed overall survival at 12 years of 54% (95% CI: 47.8, 62), although with limited numbers of patients.^11^ Another retrospective, single‐center study of the Magna Ease valve in 1126 consecutive patients reported a 78.2% survival probability at 9 years.^9^ Studies of the Trifecta valve (Abbott Laboratories) have shown comparable midterm all‐cause and valve‐related mortality.^12^, ^13^, ^14^ The Freestyle valve (Medtronic) showed similar rates of 10‐year survival to Magna Ease in a retrospective cohort analysis.^15^, ^16^ In another multivariable retrospective analysis, the Mitroflow valve (LivaNova) was associated with a higher risk of mortality compared with the Magna Ease valve at 5 years (hazard ratio 1.57 [95% CI: 1.17, 2.11], *p* < .01).^17^

### 4.2 Thromboembolic events, bleeding, and endocarditis

Early and late complications in this study were consistent with others.^5^, ^12^ Freedom from endocarditis, thromboembolism, and major bleeding at 8 years were 97.3% (95% CI: 95.1, 99.5), 86.7% (95% CI: 81.9, 91.5), and 85.1% (95% CI: 80.0, 90.1), respectively. The reported rate of bleeding events does not pose an unexpected or additional risk to patients treated with the Magna Ease valve. Most of the bleeding events had no further clinical consequences, and those observed were typical for older patients following cardiac surgery.

### 4.3 Hemodynamic performance

The mean gradient remained acceptable at 8 years: 14.8 ± 8.3 mmHg compared with 12.2 ± 5.0 mmHg at 1 year and was consistent with findings of a recent study.^9^ Although one meta‐analysis concluded gradients were lower for the Trifecta valve than the Magna and Magna Ease valves at 6 months (mean difference 4.1 mmHg; 95% CI: 3.5, 4.7; *p* < .0001),^18^ a recent large comparative analysis found a concerning decline in Trifecta hemodynamics over 5 years, with an increased rate of explant due to structural deterioration, compared with the original PERIMOUNT valve.^19^

The hemodynamic improvement seen in this study resulted in a sustained improvement in NYHA functional class for nearly two‐thirds of patients.

### 4.4 Valve durability

Freedom from SVD was 99.1% (95% CI: 97.9, 100.0) at 5 years and 90.1% (95% CI: 84.7, 95.4) at 8 years. The 8‐year rate is slightly lower than the 10‐year freedom from SVD rates with the Magna Ease valve reported by Bourguignon et al.^5^ (94.2%) and Forcillo et al.^20^ (98 ± 0.2%), or the 12‐year rates from Piperata et al. (93%).^11^ However, comparing SVD rates from different studies is challenging because of differing SVD definitions and cohort ages. Freedom from SVD with the Trifecta valve was reported to be 98.7% and 93.3% at 5 and 8 years, respectively,^13^ although a recent study demonstrated an increased risk of valve failure in the Trifecta valve compared with the Magna Ease valve at 48 months.^16^

Freedom from reintervention due to SVD was 99.1% (95% CI: 97.9, 100.0) at 5 years and 93.6% (95% CI: 89.3, 97.8) at 8 years. Rates reported for the Trifecta valve were 97.3% (95% CI: 94.7, 98.6) at 6 years.^12^, ^21^ In a propensity score‐matched analysis between the Trifecta valve and the Magna Ease valve, the Trifecta valve cohort had a significantly higher risk of repeat AVR for structural valve failure at 7 years (5.7% vs. 0%, *p* = .009).^14^ Another propensity score‐matched analysis showed significantly lower freedom from explant for the Trifecta valve compared with the PERIMOUNT valve at 5 years (95.9% vs. 98.7%, *p* < .001).^19^

In an effort to reduce SVD and improve the durability of bioprosthetic valves, Edwards Lifesciences has developed RESILIA tissue with advanced anticalcification technology, but durability data beyond 5 years with this tissue are still awaited.^22^, ^23^

## 5 LIMITATIONS

his study has some limitations. While it includes outcomes from 14 centers and used an independent CEC to adjudicate safety events, an echo core lab was not used, potentially introducing variability, as echocardiographic data were collected and evaluated by individual centers. Second, this study did not include a comparator study arm to compare these Magna Ease valve outcomes with those of other contemporary valves.

## 6 CONCLUSIONS

This is the first multicenter, prospective cohort study of the Magna Ease valve, with CEC adjudication of all safety events. The Magna Ease valve demonstrated satisfactory freedom from mortality and valve‐related complications requiring reintervention and sustained improvement in hemodynamics at 8 years. These data support the continued use of this valve, adding to the growing body of evidence that the Magna Ease valve represents a standard of performance against which other surgical valves may be compared.^7^, ^9^

## CONFLICTS OF INTEREST

*Financial support from British Standards Institute and 3R Life Sciences*: Steven Tsui. *Shareholder in Edwards Lifesciences*: Michael Rosenbloom. *Honoraria for meetings from Edwards Lifesciences*; *owns stock in Kardium Inc*.: James Abel. *Consulting fees from Edwards Lifesciences*: Axel Haverich. *Financial support from Edwards Lifesciences, Medtronic, CryoLife Inc., Ethicon, Cambridge Medical Robotics, and LSI Solutions®*: Joseph Zacharias. *Consulting fees from Edwards Lifesciences*: Gideon Cohen. *Financial support from Medtronic and owns stock in Highlife*: Rüdiger Lange. Gilbert Schorlemmer, Jeffrey Swanson, and Michael Moulton declare no conflict of interest.

## ETHICS STATEMENT

All study sites obtained Institutional Review Board (IRB) approvals: UBC‐Providence Health Care Research Institute, Vancouver, BC, Canada, #H11‐00994, 06/24/11; Providence Health and Services IRB, Portland, OR, USA, #10‐063B, 07/28/10; Sunnybrook Health Sciences Centre REB, Toronto, ON, Canada, #382‐2007, 02/21/08; Dignity Health IRB, Sacramento, CA, USA, #033588, 11/17/11; Atlantic Health IRB, Morristown, NJ, USA, #R09‐12‐004 (455058), 01/07/10; The Cooper Health System IRB, Camden, NJ, #09‐190EX, 02/05/10; Mountain Star St. Mark's Hospital IRB, Salt Lake City, UT, USA, #0221, 01/21/10; MHH Ethics Committee 30623 Hannover, Germany, #5083, 08/22/08; NHS National Research Ethics Committee, Papworth, Cambridge, UK, #08/H0304/34, 05/30/08; NHS National Research Ethics Committee, Blackpool, Lancashire, UK, #08/H0304/34, 07/23/08; Ethics Committee of the University Hospital Puerta de Hierro, Madrid, Spain, #892, 02/26/08; Ethics Committee of the Faculty of Medicine of the Technical University of Munich, Munich, Germany, #1913/07, 12/04/07; Ethics Committee of the Medical University Innsbruck A‐6020, Innsbruck, Austria, #EK 1 25.09.07, 01/29/08; and Western IRB, Olympia, WA, USA (University of Arizona, Tucson, AZ, USA), #20110574. All study participants provided informed written consent.
